# A Review of Nonanesthetic Uses of Ketamine

**DOI:** 10.1155/2020/5798285

**Published:** 2020-04-01

**Authors:** Abby Pribish, Nicole Wood, Arun Kalava

**Affiliations:** ^1^Department of Medicine, Beth Israel Deaconess Medical Center, Boston, MA, USA; ^2^Department of Medicine, Cleveland Clinic, Cleveland, OH, USA; ^3^Department of Anesthesiology, University of Central Florida College of Medicine, Orlando, FL, USA

## Abstract

Ketamine, a nonselective NMDA receptor antagonist, is used widely in medicine as an anesthetic agent. However, ketamine's mechanisms of action lead to widespread physiological effects, some of which are now coming to the forefront of research for the treatment of diverse medical disorders. This paper aims at reviewing recent data on key nonanesthetic uses of ketamine in the current literature. MEDLINE, CINAHL, and Google Scholar databases were queried to find articles related to ketamine in the treatment of depression, pain syndromes including acute pain, chronic pain, and headache, neurologic applications including neuroprotection and seizures, and alcohol and substance use disorders. It can be concluded that ketamine has a potential role in the treatment of all of these conditions. However, research in this area is still in its early stages, and larger studies are required to evaluate ketamine's efficacy for nonanesthetic purposes in the general population.

## 1. Introduction

Ketamine has been used as an anesthetic drug for over 65 years [1]. An enantiomeric, lipid-soluble phencyclidine derivative, ketamine is one of the most commonly used drugs in anesthesia. As a nonselective NMDA receptor antagonist, it has equal affinity for different NMDA receptor types. NMDA is a subgroup of ionotropic glutamate receptors, along with AMPA and kainite. Ketamine is inexpensive and therefore widely used in developing countries. It additionally has particular utility for anesthesia induction in hemodynamically unstable patients [2].

**Figure 1:**
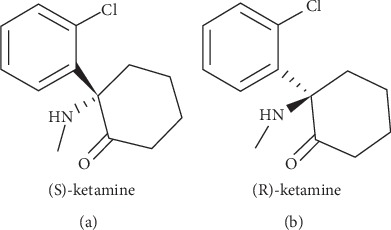
The structure of (a) (S)-ketamine and (b) (R)-ketamine.

Ketamine administration has long been known to mediate a wide variety of pharmacological effects, including dissociation, analgesia, sedation, catalepsy, and bronchodilation. Though ketamine is known most widely for its anesthetic properties, recent research has uncovered multiple novel uses for this drug, including neuroprotection, combatting inflammation and tumors, and treatment of depression, seizures, chronic pain, and headache [3–5]. Racemic ketamine, a mixture of (S)- and (R)-ketamine (Figure 1), is commonly used in this research, though both (S)-ketamine and (R)-ketamine alone are also subjects of study. While (S)-ketamine carries roughly 3- to 4-fold greater potency as an anesthetic, it also carries a greater risk of psychotogenic side effects [6]. However, ketamine has an extensive side-effect profile and a potential for abuse that cannot be ignored, which has historically led to its avoidance in favor of other agents, and its safety is an area of ongoing research [3]. Additionally, there are a variety of adverse reactions that have been associated with ketamine use which must be considered, including self-resolving sinus tachycardia, neuropsychiatric effects, abdominal pain, liver injury, and dose-dependent urogenital pathology including ulcerative cystitis [7–9]. Currently, there are roughly 800 or more clinical trials exploring aspects of nonanesthetic uses of ketamine registered on ClinicalTrials.gov, illustrating the extensive ongoing interest in this area.

The nonanesthetic clinical uses of ketamine have been the focus of extensive recent research, some of the most applicable and prevalent of which are explored here. For this scoping study, we sought to utilize the Arksey and O'Malley methodological framework to provide a broad overview of the field, with attention to ongoing research and current knowledge gaps [10]. Relevant literature from 2010 through the present was queried through the MEDLINE, CINAHL, and Google Scholar databases. Keywords included “ketamine” combined with terms including “non-anesthetic uses,” “depression,” “headache,” “neuroprotection,” “pain,” “pain syndromes,” “chronic pain,” “alcohol use disorder,” “substance use disorder,” and “seizure.” Sentinel research from prior to 2010 was also incorporated. Relevant original articles including randomized trials, retrospective studies, review articles, case reports, and preclinical animal studies were included. This paper will discuss some of the most common and promising nonanesthetic uses of ketamine, including its utility in the treatment of depression, pain syndromes including headaches, neurologic disorders including seizures, and alcohol/substance use disorders.

## 2. Ketamine and Depression

Despite the high prevalence of depression, which affects roughly 1 in 5 people over their lifetime, currently available pharmacologic treatments, the most commonly utilized of which are selective serotonin reuptake inhibitors (SSRIs), have limited efficacy [11]. SSRIs achieve adequate effect in as little as 30% of patients [12], while having a high burden of side effects ranging from nausea and headaches to weight gain and sexual dysfunction [13]. Pharmacologic treatment of depression has also historically been limited by the fact that conventional antidepressants typically take weeks to reach effect [14]. Nearly all antidepressants target monoaminergic systems, and research on new molecular targets (including corticotropin-releasing factor 1 antagonists, neurokinin 1 antagonists, and vasopressin V1b antagonists) has not yet led to alternative treatments [15]. Depression is known to be associated with alterations in glutamatergic neurotransmission and dysfunctional activity of the resting state network [16]. Additionally, depression is thought to be caused by enhanced subcortical and limbic activity, which affects cognition and emotion regulation [15]. Ketamine offers a promising alternative to conventional antidepressants due to its rapid onset and apparent efficacy. More broadly, ketamine appears to have efficacy in treating multiple internalizing disorders including depression, anxiety, and obsessive-compulsive disorder [17–19].

Ketamine is thought to affect these brain areas directly through modification of glutamatergic neurotransmission [20], although it has also been shown to mediate its effects through modulation of dopaminergic neurotransmission [21] and serotonergic neurotransmission [20]. Ketamine also indirectly acts through several other neurochemical pathways. It induces upregulation of the mammalian target of rapamycin (mTOR) pathway, shifting activity away from subcortical and limbic regions and toward the medial and lateral prefrontal cortex [15], and has the potential to reverse the mTOR signaling pathway impairment that is seen in major depressive disorder (MDD) [22]. Ketamine additionally upregulates the expression of glutamate transporters, specifically EAAT2 and EAAT3, in the rat hippocampus [23]. Modulation of hippocampal plasticity is another mechanism by which ketamine is thought to mediate its antidepressant effects [14], the mechanism of which may be related to EAAT3 regulation of AMPA receptor trafficking and redistribution [23].

A single subanesthetic dose (0.5 mg/kg) of intravenous (IV) ketamine hydrochloride has been shown to have a rapid antidepressant effect, which begins as early as 2 hours after ketamine administration, peaks at 24 hours, and lasts for up to 7–14 days [24, 25]. This effect has been noted in both unipolar and bipolar depression [26], although effect duration may be shorter in patients with bipolar disorder [27]. Promisingly, efficacy from ketamine is seen in people with treatment-resistant depression, who have failed multiple antidepressant regimens [28, 29]. In one study of 67 patients (including 45 women), IV ketamine administered at 0.5 mg/kg twice per week has been shown to achieve rapid-onset and sustained antidepressant effect for a 15-day period [30].

Broadly there are 2 generations of studies evaluating ketamine for unipolar depression: (1) studies on safety/efficacy of one subanesthetic dose of IV ketamine and (2) studies on alternate drug delivery routes, MDD relapse prevention, and mechanistic analysis [15]. The first study on single-dose IV ketamine in seven patients with mood disorders was published in 2000 by Berman et al. and found a significant but transient improvement of depression severity with a single subanesthetic dose of IV ketamine (0.5 mg/kg) [31]. While the improvement to depression symptoms was transient, this improvement did exceed the elimination half-life of ketamine [15]. A larger replication study with 18 subjects was published by the Intramural Research Program of the National Institute of Mental Health (NIMH) in 2006 and also found that subanesthetic ketamine (0.5 mg/kg) has a significant antidepressant effect [17]. This clinical trial has largely been credited with launching the field of research into ketamine's antidepressant effects [32]. Multiple open-label case series have demonstrated similar results with a single ketamine infusion [33]. Subsequent research has shown that a regimen of serial IV ketamine (0.5 mg/kg) infusions achieves a greater response rate, without more significant side effects [33, 34]. Other routes of ketamine administration have also been examined. For example, intranasal ketamine hydrochloride (50 mg) has been shown to mediate an antidepressant effect, though the magnitude of the effect may be less than that of IV ketamine [35]. Intranasal esketamine (which is the S(+) enantiomer of ketamine) in combination with an oral antidepressant was recently approved by the Food and Drug Administration for the treatment of treatment-resistant depression, though the long-term effects of this regimen remain preliminary [36]. However, when used for its antidepressant effect in mice, (R)-ketamine appears to have more potent and persistent effects than (S)-ketamine, as well as no psychotomimetic side effects [6].

IV ketamine may have increased utility in specialized populations, such as the military, cancer patients, and patients with Alzheimer's disease. In active duty military populations, long-term psychiatric admission for suicidality may create unique problems including separating the patient from his or her support network and leading to administrative obstacles in returning to duty [37]. In one study on 10 soldiers in the United States, a single dose of IV ketamine (0.2 mg/kg) was found to significantly decrease suicidality and hopelessness [37]. Ketamine appears to be a rapidly efficacious antidepressant and antisuicidal pharmacologic agent which may be well suited for this particular population and others in which long-term psychiatric hospitalization creates significant challenges, though it is possible that the particular applications for the US military may not translate to broader military use around the world. IV ketamine (0.5 mg/kg) has also demonstrated utility in treating acute-onset depression and suicidal ideation in one study of 39 newly diagnosed cancer patients [38]. Furthermore, ketamine may have unique utility in treating depression associated with Alzheimer's disease, as ketamine appears to have neuroprotective properties against soluble amyloid-beta protein-mediated toxicity, according to one study utilizing 15 mg/kg intraperitoneal ketamine in mice [39].

The rapid-onset antisuicidal properties of ketamine are possibly mediated by enhancing neuroplasticity [15, 24, 40]. This effect is even seen in patients who are nonresponders to the antidepressant effect of ketamine [41]. Improvement in suicidal ideation occurs as early as within 40 minutes of subanesthetic dose ketamine administration (0.5 mg/kg) and may last as long as 10 days, according to one study of 57 patients [42]. While change in depressive symptom severity correlates with change in suicidal ideation, even when depressive symptom severity is controlled for, the antisuicidal effect of ketamine persists [43]. Ketamine has been demonstrated to effectively treat anhedonia independent of depressive symptoms, and this effect can last up to 14 days [44]. It has been theorized that reducing anhedonia is the mechanism by which ketamine reduces suicidal thoughts [45]. Ketamine has also been shown to have anxiolytic and procognitive effects in a rat model of depression, using doses between 5 and 30 mg/kg [46]. Additionally, when used as the anesthetic during electroconvulsive therapy (ECT), ketamine decreases Hamilton Depression Rating Scale scores earlier and more significantly than when propofol is used [47]. However, while meta-analysis of 16 articles with 346 patients has demonstrated superior treatment effect in patients with depression who receive ketamine in ECT over other anesthetics, these patients were also noted to have more side effects and longer recovery times [48].

While ketamine has many clinically promising features, it has a number of drawbacks to consider. One of these is effect duration: the antidepressant effect of ketamine lasts on average only 1-2 weeks [49]. There is substantial variation in the duration of treatment response, with many patients reporting less than 1 week of depressive symptom improvement from a single ketamine infusion [27]. However, a ketamine maintenance infusion regimen, in which infusions of ketamine 0.5 mg/kg are administered up to every 2 weeks, has shown promising results in a study of 8 patients [50]. Due to poor bioavailability resulting from high first-pass hepatic metabolism, ketamine is typically administered by injection, which is a drawback for medications that require ongoing dosing [21, 51]. IV administration every 2-3 days requiring hospital or clinic visits is impractical. However, alternative routes of administration offer promising alternatives (for example, very-low-dose sublingual ketamine has been shown to improve mood, cognition, and sleep, when 10 mL of a 10 mg/mL solution is administered sublingually for 5 minutes and swallowed) [24]. Another drawback of ketamine is that, as a derivative of phencyclidine (PCP) [11], ketamine causes a transient increase in psychotomimetic symptoms [15, 41] and dissociative symptoms, though these return to baseline by 4 hours posttransfusion [41]. Ketamine has abuse and addiction potential [1, 37] and causes cognitive deficits, which have been shown to be reversible with cessation of ketamine use [15]. However, concerningly, chronic recreational ketamine use has also been shown to produce cognitive and affective deficits including depression [52], which raises concern about the use of ketamine as an option for long-term antidepressant therapy. Other potential adverse effects of ketamine include transient tachycardia and hypertension [1]. There are also concerns about neurotoxicity, bladder toxicity, and tolerance with repeated ketamine infusion use [34].

As a result of these drawbacks, many clinicians and researchers view IV ketamine infusions not as an end-all replacement for conventional antidepressants, but a promising new direction for antidepressant therapy that warrants further research and an effort to develop “ketamine-like” drugs that do not carry the side effects that currently limit the use of ketamine [1, 25, 32]. For example, the possible antidepressant effects of other glutamatergic modulators, including riluzole, dextromethorphan, nitrous oxide, and GLYX-13 (rapastinel), are currently being examined [53].

## 3. Ketamine and Pain Syndromes

Ketamine has been widely used to manage acute and chronic pain, both alone and as an adjunct to opiates. The primary analgesic mechanism of ketamine is through NMDA receptor antagonism, though ketamine has also been shown to act on opioid, nicotinic, and muscarinic receptors. Ketamine's anti-inflammatory qualities may also contribute to its efficacy in pain relief [54, 55]. While ketamine's effect on acute pain is driven primarily by inhibition of NMDA receptors and prevention of wind-up, ketamine is thought to mediate its effect on chronic pain through desensitization of upregulated NMDA receptors [56–58]. Routes of ketamine administration for analgesia include parenteral, oral, sublingual, topical, and intranasal [54]. It appears that administration of high-dose ketamine over a short time course (42–480 mg daily for 1–10 days) produces analgesia more effectively than lower doses for longer durations (such as 18 mg daily for 90 days) [59]. The level of evidence and consensus for the utility of ketamine in pain management varies between types of pain.

### 3.1. Acute Pain

Ketamine appears to reduce analgesic requirement in the setting of acute pain. For example, in 160 patients undergoing cesarean section, a single postoperative intravenous ketamine bolus (0.25 mg/kg) was shown to reduce the severity of postoperative pain and decrease analgesic requirements [60]. As a result, ketamine can prevent opioid tolerance [61] and may reduce the rate of opioid-induced hyperalgesia following surgery [62], while also mitigating adverse effects linked to opiates such as respiratory suppression, oversedation, and hypotension [63]. In addition its opioid-sparing effects, ketamine has been shown to reduce nausea and vomiting in the perioperative period at doses of <0.5 mg/kg [64]. While ketamine has generally been shown to reduce intraoperative opioid requirements in both opioid-naïve and opioid-dependent populations [62, 65], this is somewhat controversial. Some studies have demonstrated decreased average pain scores when continuous ketamine (0.2 mg/kg/hour) is used intraoperatively, but no decrease in overall opioid requirement [66]. Furthermore, other studies show no difference in postoperative pain levels or postoperative opioid requirement in postsurgical patients when ketamine is used, including several studies that demonstrated no benefit to the use of ketamine infusion in patients undergoing spinal surgery [67, 68].

Because of its efficacy in treating acute pain, ketamine has utility in the acute care setting. Ketamine has well-established utility in the emergency department (ED) as short-term analgesia for indications such as acute long bone fractures, trauma victims, and opioid-dependent patients with acute pain, which in one study was administered as ketamine 15 mg IV once followed by a continuous ketamine infusion at 20 mg/hour for 1 hour [69]. When used alone for pain management in the ED setting, low-dose ketamine (<1 mg/kg) provides comparable pain relief to opiates, with the benefit of producing less respiratory depression [70]. Ketamine has also been shown to decrease opioid consumption for acute pain in ED patients, in a study of 30 patients with severe pain where ketamine 15 mg IV and hydromorphone 0.5 mg IV were administered together [63].

### 3.2. Chronic Pain

The role of intraoperative ketamine in the reduction of chronic postoperative pain development is unclear. Some reports suggest that ketamine decreases the rate of chronic postoperative pain when administered as a 0.15–1 mg/kg preincisional loading dose followed by intraoperative infusion [71], and intravenous ketamine has been shown in meta-analysis of 40 papers including 1388 participants to significantly reduce chronic pain incidence following certain types of surgery [72]. This effect may be mediated through a reduction in primary and secondary hyperalgesia in the postoperative period, which decreases the incidence of chronic pain [73]. However, there appears to be a reduction in acute pain but not chronic pain development following amputation, thoracotomy, or mastectomy with the use of ketamine as a coanalgesic agent [74]. Epidural ketamine and intravenous ketamine have not been shown to decrease the incidence of development of chronic postthoracotomy pain [75–77]. Additionally, meta-analysis has also not shown intravenous and epidural ketamine to significantly reduce the rate of persistent postsurgical pain (PPSP) at three or six months [78].

There is moderate evidence that ketamine effectively reduces chronic noncancer pain [59]. A recent systematic review and meta-analysis of 7 studies showed short-term analgesic benefit from IV ketamine in patients with chronic pain, which appears to occur in a dose-response relationship [79]. In a study of 49 patients, ketamine infusion was shown to decrease visual analog scale (VAS) scores in patients with intractable chronic pain, in whom ketamine 0.5 mg/kg was administered over 30–45 minutes, followed by either continuation at this dose in subsequent infusions every 3-4 weeks, or increase in the dose up to the highest tolerated dose providing analgesia [80]. Daily oral ketamine (up to 64 mg/day) has also been shown to be safe and opioid-sparing in patients with chronic pain [81]. The combination of subcutaneous ketamine infusion and sublingual ketamine lozenges appears to reduce opioid use in patients with chronic nonmalignant pain [82]. In one retrospective study of 51 patients with refractory chronic pain, oral ketamine treatment (starting at 0.5 mg/kg/day, then increased in 15 to 20 mg increments as needed) led to the resolution of pain in 44% of patients, reduced opioid requirements by an average of 62%, and was ineffective in only 22% of patients [83]. These results are especially promising because of the limitations of currently available treatments for chronic pain, with only 30–40% of patients with chronic pain achieving adequate to good relief [84]. Ketamine infusions (administered as infusions of 0.1–0.3 mg/kg/hour for 4–8 hours/day, up to 16 hours over three consecutive days) have also been shown to significantly reduce pain intensity in children and adolescents with chronic pain, with the largest benefit seen in patients with CRPS [85]. However, the utility of ketamine in treating chronic pain is not universally accepted. For example, in one study of 36 patients, ketamine was shown not to improve long-term pain scores in patients who take chronic opiates, or to effectively reduce opiate requirements [86].

The utility of ketamine has been validated in neuropathic pain [87], especially in complex regional pain syndrome (CRPS). CRPS causes significant morbidity, and 80% of patients with CRPS are severely disabled [88]. Many patients with CRPS are unresponsive to traditional therapeutic approaches, and ketamine has been shown to reduce pain levels in some of these treatment-refractory patients [89]. When studied in mice, ketamine (administered subcutaneously at a dose of 2 mg/kg/day for 7 days) appears to decrease nociceptive sensitization in the chronic stage of CRPS, but not the acute stage [55]. When CRPS type 1 (CRPS-1) alone was studied using a cohort of 10 patients, S(+)-ketamine infusion (using the following regimen per 70 kg: min 0–5: 1.5 mg, min 20–25: 3.0 mg, min 40–45: 4.5 mg, min 60–65: 6.0 mg, min 80–85: 7.5 mg, min 100–105: 9.0 mg, and min 120–125: 10.5 mg) appears to reduce pain levels for 10 weeks or longer, therefore demonstrating a disease-modulatory role [56]. Despite the efficacy of ketamine as an analgesic in this population, functional improvement of affected limbs was not shown in one study of 5 patients who received anesthetic doses of ketamine over 10 days [90]. Though ketamine appears to be safe and effective in treating CRPS, further studies are warranted to evaluate dosing, timing, and routes of administration of ketamine for optimal efficacy in CRPS treatment [91]. For example, 10% ketamine cream applied three times daily in combination with oral palmitoylethanolamide has been shown in a case report to effectively treat refractory CRPS pain [92], but larger, controlled studies are warranted.

There are additional chronic pain conditions in which a small number of studies have shown promising results from ketamine, and further research is warranted. Ketamine infusions appear to be effective as an adjunct with gabapentin for managing chronic neuropathic pain in spinal cord injury patients, with a duration of efficacy lasting 2 weeks after infusion termination, according to a study in 40 patients, who received 80 mg IV ketamine over 5 hours daily for 1 week and gabapentin 300 mg 3 times daily [93]. In patients with phantom limb pain, ketamine also appears to mediate short-term analgesic effects [94]. S-ketamine has been shown to reduce chronic pancreatitis pain in a study of 10 patients when administered as an infusion of 2 *μ*g/kg/min for 3 hours, though this effect disappeared following the end of infusion [95]. Ketamine may also have utility in scenarios where opioids alone often have inadequate efficacy, including vasoocclusive episodes in patients with sickle cell disease. Few studies have examined the role of low-dose ketamine in the treatment of sickle cell pain, though the majority of reported cases have shown that ketamine effectively reduces pain intensity and opioid requirements in patients with sickle cell pain [96]. The data on this topic are limited, and further studies are warranted to validate this finding [97]. Additionally, since pain disorders are highly correlated with suicidal ideation and attempts, the antisuicidal properties of ketamine may make ketamine a useful treatment option in patients with concomitant pain and suicidal ideation [40].

Though ketamine has anecdotally been reported to effectively treat cancer pain, when studied systematically, ketamine has not been found to be useful in the treatment of pain from advanced cancer as an adjunct to opioids, though difficulty in designing studies in the context of palliative care may contribute to these results [98, 99]. Ketamine can be considered as an adjuvant therapy in patients with cancer who have failed standard therapy, though optimal dosing is unclear [100].

### 3.3. Headache

Chronic migraine affects 1% of the population within the United States, creating a significant economic burden, and treatment options for refractory cases are limited [101, 102]. Due to its efficacy in the treatment of chronic pain, it has been hypothesized that ketamine might be a useful addition to headache and migraine control regimens. For example, while triptans effectively relieve acute migraine pain in 43–76% of cases [103], ketamine could play an important role in pain control for triptan nonresponders. Ketamine could also play a role in migraine management for patients in whom triptans are contraindicated, such as patients with cardiovascular diseases. The actions of ketamine on glutamate NDMA binding sites at the level of the secondary somatosensory cortex, insula, and anterior cingulate cortex have been associated with modulation of affective pain processing and the decrease of allodynia and central sensitization. These effects associated with chronic pain might also be the basis of the mechanism of effect on headache pain [101, 104]. It is useful to take these mechanisms into account when considering memantine, a noncompetitive glutamatergic NMDA antagonist, which has been previously shown to be an effective treatment for chronic and refractory migraine [101, 105].

Minimal evidence exists surrounding the use of ketamine in chronic headache treatment. Individual cases suggest that ketamine administered IV (using an initial infusion rate of 0.1 mg/kg/hour, then increased by 0.1 mg/kg/hour every 3-4 hours until a goal pain score 3/10 was reached and maintained for 8 hours, then downtitrated) in inpatient management of refractory migraine consistently reduces short-term pain severity, although no chronic relief has been observed [101]. A large review including 77 patients has demonstrated similar results, with intravenous ketamine administration (starting at an infusion rate of 0.1 mg/kg/hour, increased as needed at 6-hour intervals to a maximum infusion rate of 1 mg/kg/hour) causing acute but not long-term improvement to refractory headache [106]. When considering alternate methods of delivery, randomized controlled trials and case studies of intranasal ketamine's effects on migraine with aura have demonstrated that 25 mg intranasal ketamine reduces the severity, and in some cases the duration, of the associated aura [107, 108]. This further reinforces the potential of the use of drugs with action on glutaminergic pathways, such as ketamine, as headache modulators [107].

Ketamine has also been investigated in combination with other drug regimens. Magnesium sulfate, which binds to NMDA channels, might be administered concomitantly with ketamine to produce a heightened effect. When given intravenously to 2 chronic cluster headache patients, this combination (ketamine 0.5 mg/kg over 2 hours and magnesium sulfate 3000 mg over 30 minutes) was shown to produce immediate pain relief, a decrease in suicidal ideation, and a decrease in attack frequency and intensity for up to six weeks [109]. Evidence exists that levels of kynurenic acid, an NMDA receptor antagonist, are decreased in cluster headache patients, providing further support for the theory that NMDA receptors are overactive in these patients and that a focus on therapeutic options targeting these receptors is warranted [109, 110].

Unfortunately, there are also several pieces of contradictory evidence against the use of ketamine for the treatment of primary headache [111]. Small randomized studies have shown no improvement in acute headache pain outcomes with IV ketamine (0.2-0.3 mg/kg) when compared to both placebo and prochlorperazine, while also inducing increased side effects [111–113]. Additionally, most investigations of this use of ketamine are reported as small case series, and further study is required in order to make informed conclusions on the efficacy of ketamine in the treatment of headache. The current body of literature has led to the conclusion by some experts that there is not sufficient evidence for the widespread use of ketamine in headache patients [111, 114].

### 3.4. Drawbacks

Ketamine has multiple drawbacks as a treatment for pain. Ketamine may have limited utility as a treatment for chronic pain syndromes given the potential risks associated with repeated IV administration of ketamine, including its neurotoxicity and potential to impair long-term memory [115]. While these risks have not yet been formally studied in a controlled fashion, the effect of frequent (defined as more often than twice per month) recreational ketamine use was shown in a study of 37 patients to cause long-lasting impairments in episodic and semantic memory [116]. Furthermore, both sensitization and tolerance are possible consequences of repeated ketamine use, and while the duration required to notice these effects from intermittent ketamine use has not been extensively studied in humans, in mouse studies sensitization has been shown to occur over the course of weeks and is clearly evident by 5 weeks of weekly administration of intraperitoneal ketamine (20 mg/kg or 50 mg/kg, in mice) [117]. Ketamine also has been known to cause hepatic toxicity due to mitochondrial impairment, urological toxicity including ulcerative cystitis, and immediate risks including tachyarrhythmias, hallucinations, and flashbacks [88, 118, 119]. Psychedelic effects are also associated with ketamine [59], and benzodiazepine coadministration may be required to treat its psychosis-like effects [58].

Most studies on the utility of ketamine in pain management have small sample sizes, and treatment effect may therefore be overestimated [72]. This suggests the need for larger trials evaluating the use of ketamine in pain control. Further research is also required to characterize the role of ketamine in cancer-related pain [120], including the role of oral ketamine in palliative care [121]. Furthermore, while ketamine infusions have been well studied, alternative routes of ketamine administration have been evaluated less extensively. For example, open studies of 2% topical ketamine preparations have suggested a therapeutic effect on chronic pain without adverse effects locally or systemically, though further research is needed to elucidate its efficacy [122]. The S-enantiomer of ketamine also appears to have a two- to threefold more potent analgesic effect than (R)-ketamine [123], and the utility of using (S)-ketamine alone as a treatment for pain warrants additional study.

## 4. Neurologic Applications of Ketamine

### 4.1. Neuroprotection

In addition to mediating anesthetic effects, the noncompetitive antagonism of NMDA by ketamine has recently been postulated to play a role in neuroprotection. Ketamine was previously thought to increase intracranial pressure (ICP) [4] and therefore would be contraindicated in cases where ICP may already be elevated (such as trauma and neurosurgical patients). This conclusion was based on a few small studies with limited scope, but did result in an FDA package insert warning [124]. Several more recent studies challenged and disproved this theory [4, 124]. These reports of cases linking ketamine induction to elevated ICP may not have adequately taken ventilation into account; in one case of reported elevated ICP after ketamine induction, the patient was spontaneously breathing after induction, and ICP was noted to decrease dramatically with initiation of manual hyperventilation [125]. Therefore, hypercarbia is the more likely underlying cause of ICP elevation rather than use of ketamine induction, and in patients who undergo ketamine induction and normocarbia is maintained using mechanical ventilation, rise in ICP is not seen [4].

Several mechanisms of action behind ketamine's neuroprotective qualities have been proposed. Ketamine has anti-inflammatory properties and is thought to reduce microglial activation and reduce cytokines TNF and IL-6, although studies have not been able to prove any differences in plasma inflammatory markers after ketamine administration [5, 124, 126]. It is known that unlike other anesthetic drugs including propofol, ketamine does not provide neuroprotection via inhibition of TLR-4-NF-*κ*B-dependent signaling [127]. Through its NMDA inhibition, ketamine reduces glutamate excitotoxicity by preventing excitatory amino acid receptor stimulation, and this reduction has been proven through the use of MRI, in one study of 24 infants [124, 126]. Excitotoxicity, defined as the excessive stimulation of neurons causing neuronal injury, has been suggested as the underlying process behind several types of central nervous system pathology [124]. Ketamine reduces neuronal death and injury through the blockade of calcium entry into vulnerable immature neurons [126, 128]. NMDA receptor activation is also thought to cause the loss of mitochondrial membrane potential and apoptosis through cAMP response element binding protein shutoff, a process that NMDA inhibition by ketamine would also prevent [124]. Finally, it is well documented that ketamine protects against ischemic injury by reducing cell swelling and preserving cellular energy following anoxia-hypoxia injury, while also increasing neuronal viability and preserving cellular morphology [129–131]. It is hypothesized that inhibition of P-CREB dephosphorylation in the infarct area by low-dose ketamine is responsible for a decrease in infarct volume, edema ratio, and neurologic deficit [132]. These are all processes known to be induced by cerebral injury such as stroke and trauma, which gives ketamine promising clinical implications [4].

Ketamine appears to be beneficial in neuroprotection following multiple types of neural injury. Studies have shown that ketamine reduces focal ischemia and hemorrhagic necrosis volumes as well as chronic cerebral hypoperfusion [4, 5, 133–135]. In animal studies, outcomes following incomplete cerebral ischemia were improved with ketamine administration, thought to be related to reduced plasma catecholamine levels [5]. Additionally, ketamine causes an increase in blood flow regionally and globally and reduces resistance in the cerebrovasculature [4, 136, 137]. Ketamine provides some measure of cardiovascular stimulation as well, which may contribute to cerebral perfusion [138]. For example, ketamine might have utility as a hemodynamic agent in traumatic brain injury (TBI) patients with hypovolemia, as it is well documented that ketamine can cause an elevation in heart rate, systolic blood pressure, and cardiac index [124]. Studies have shown that ketamine also inhibits spreading depolarizations, which cause depression of neuronal activity. These slow potential changes propagate in brains with previously existing ischemic damage to cause or increase damage, and their prevention could improve outcomes in TBI, subarachnoid hemorrhage, and malignant stroke cases [124, 139, 140]. In TBI specifically, which involves increased inflammation, autophagy, edema, and ischemia, ketamine produces several beneficial effects. At subanesthetic doses in animal models, it prevents IL-6 and TNF-*α* release, reduces deficits in dendrites, and possibly activates the mTOR signaling pathway to downregulate autophagic protein production [141]. This has been translated to clinical TBI research: in one study of 115 brain-injured patients, ketamine administration (with a median dose of 200 mg) was found to reduce the occurrence of the isoelectric spreading depolarizations that are seen in traumatized human cortex [139]. In another study of 66 patients with aneurysmal subarachnoid hemorrhage, (S)-ketamine infusion (with a mean dose of 2.8 ± 1.4 mg/kg/hour) significantly decreased the incidence of spreading depolarizations [142]. While the role of ketamine in spinal cord injury has been shown in animal models [143, 144], this has not yet been translated to human research.

Ketamine's neuroprotective effects have also been proven clinically through functional assessment. In human cardiac surgery patients, single-dose ketamine (0.5 mg/kg) administration at surgery induction has been associated with reduced postoperative delirium and cognitive dysfunction, results which are attributed to the reduction in systemic inflammation secondary to ketamine usage [4, 5, 138]. In animal models, ketamine has reduced impaired cognitive behavior caused by cell death in the cortex and hippocampus [129, 145] and has attenuated functional deficits in memory and behavior caused by TBI [141].

While multiple studies support ketamine's potential for neuroprotection, some others provide inconclusive evidence. Ketamine's effects on neurologic injury following cardiopulmonary bypass have been studied in both adults and children, with no resulting evidence for either neuroprotection or neurotoxicity [126, 146]. A review of neuroprotective agents administered in the perioperative period reveals that intravenous ketamine is associated with no significant difference or change in new postoperative cognitive deficits or mortality and concludes that there is currently not enough evidence to show that ketamine has a neuroprotective effect [138, 146, 147].

Conversely, ketamine has also been shown to cause apoptotic cell death in neurons, specifically in the frontal cerebral cortex and hippocampal region, as well as long-term deficits in cognitive processing [124, 128, 139, 148–150]. In animal models, ketamine at anesthetic doses is observed to collapse cortical neuron growth cones [151]. Cell injury caused by ketamine seems to be dose- and time-dependent [129, 150], secondary to an induced aberrant cell cycle reentry leading to apoptosis [129, 152]. This window of neurotoxicity seems to be focused during early brain development and significant synaptogenesis [149], particularly toward the end of pregnancy and in the early postpartum period. In mice and rats, the window of greatest vulnerability to neurotoxic agents is the first 2-3 weeks after birth, and in humans, the time of greatest vulnerability spans from midgestation to 2-3 years of life [149]. In human forebrains, NMDA receptor expression peaks during gestational weeks 20–22, which coincides with the beginning of the brain growth spurt which lasts into the postnatal period [153]. In neonatal mice, high-dose ketamine causes severe degeneration of parietal cortical cells with resultant learning and memory deficits at 2 months [154]. Long-term neurofunctional outcomes are also impaired after three daily doses of ketamine, with increased numbers of apoptotic cells in the hippocampus and later defects in learning and memory [149, 155]. Promisingly, one study has shown the potential of ketamine to counteract its own neurotoxic effects by inducing the production of the activity-dependent neuroprotective protein (ADNP); pretreatment with a subanesthetic dose of ketamine before sedation might upregulate the production of this protein and provide a neuroprotective effect in rats [151]. Other approaches to mitigating the risk of ketamine-induced neuronal apoptosis are being investigated; for example, clozapine has been shown to improve the viability of mouse neuronal stem cells that are exposed to ketamine [156].

The juxtaposition of ketamine's neurotoxic and neuroprotective effects provides an interesting conundrum. These effects seem to vary not only by acute dosage and cumulative usage over time, but also by the state of the brain (absence versus presence of noxious stimuli) during the time of ketamine introduction [149]. Some have concluded based on the existing evidence that the neuroprotective effects of ketamine are largely dependent on the use of lower doses, as higher doses can result in ketamine-induced toxicities [129, 157]. Further study is clearly required, specifically in the areas of ongoing brain development in pediatric populations as well as in the time period surrounding surgery [149]. Additionally, further study is required in human models, as much of the current evidence is based on animal models. Ketamine clearly has a great amount of promise as a neuroprotective agent, although the exact parameters of its use require further elucidation.

### 4.2. Seizures

While benzodiazepine monotherapy is the preferred treatment for isolated seizures and there is no broadly accepted role for ketamine as a treatment for isolated seizures, ketamine has the potential to play a role in the treatment of status epilepticus (SE), in which seizure activity persists for longer than 5 minutes [158]. The utility of ketamine in treating status epilepticus may be explained by the fact that sensitivity to GABA agonists decreases with seizure duration, but this is not as profound with NMDA receptor antagonism [159], and synaptic NMDA receptors may even be upregulated in prolonged seizures [160] and therefore represent an ideal pharmacologic target. Ketamine also appears to reduce glutamate uptake and may be protective against glutamate-induced neurotoxicity in the setting of seizure [161]. Ketamine appears to work synergistically with benzodiazepines to treat SE, and dual therapy using midazolam and ketamine (4.5 mg/kg midazolam with 45 mg/kg ketamine) has been shown to treat SE more effectively than either agent alone [162]. Furthermore, ketamine (10 mg/kg) in combination with a benzodiazepine (diazepam 1 mg/kg) and either valproate (30 mg/kg) or brivaracetam (10 mg/kg) has been shown to be both more effective and less toxic than benzodiazepine monotherapy for the treatment of SE [163].

Ketamine has a promising role in the treatment of refractory status epilepticus (RSE), which is defined as seizure activity that does not respond to two antiepileptic drugs at appropriate doses, and is seen in around 30% of cases of status epilepticus [164, 165]. IV Ketamine appears to effectively terminate RSE (when administered as a 0.5 mg/kg IV bolus followed by a continuous infusion gradually uptitrated to 1.5 mg/kg/hour) [166], and while most studies evaluating the use of IV ketamine in status epilepticus are in adults, ketamine also appears to be both safe and effective in children with refractory status epilepticus, at a mean dose of 40 *μ*g/kg/minute [167]. Since RSE is conventionally treated using anesthetics which require intubation, utilizing ketamine in the treatment of RSE can prevent the need for intubation and spare patients the associated risks [168]. RSE carries significant morbidity and mortality, with up to 90% of individuals with RSE suffering severe morbidity and up to 19% of individuals with SE lasting greater than 30 minutes experiencing death [169], making novel treatment options like ketamine valuable. Ketamine infusion (with a maximum dose range of 25–175 *μ*g/kg/minute) with or without propofol has also been shown in a study of 67 patients to effectively control superrefractory status epilepticus (SRSE), in which seizures persist for at least 24 hours after anesthetics are initiated [170]. Ketamine (either as a 1.1–4 mg/kg bolus or as 1.0-1.1 mg/kg/hour infusion) also appears to be protective in cases with both RSE and traumatic brain injury, according to a retrospective review of a cohort of 11 patients [165].

In the context of chemical warfare, ketamine may have a role in neuroprotection and reducing neuroinflammation induced by organophosphorus nerve agents, which are known to cause seizures, status epilepticus, and brain damage. Ketamine in combination with atropine, with or without a benzodiazepine, appears to have utility in reducing the effects of organophosphorus nerve agents including soman, which could have utility in field conditions [171]. A study in guinea pigs exposed to soman showed that (S)-ketamine and atropine provided comparable protection against death and seizure-related brain damage, but at doses 2-3 times lower than racemic ketamine and atropine [172].

While a promising treatment for RSE and SRSE, ketamine has several notable drawbacks. It appears that ketamine alone may not be an effective treatment for status epilepticus that has lasted for over one hour [158]. Adverse reactions to ketamine have also been reported, including psychiatric symptoms like hallucinations and delirium, increased saliva secretion, and arrhythmias, though these are noted to be treatable and self-limited [173]. Major complications have not been reported [174]. Ketamine-induced neurotoxicity has been described, primarily using animal models [164]. Cerebellar syndrome including cerebellar atrophy has been reported with high-dose ketamine [175].

There is limited prospective data on the treatment of SE and RSE using ketamine, and this topic warrants further research [176, 177]. While a racemic mixture of (S)- and (R)-ketamine is typically used, it has been shown that (S)-ketamine is more rapidly eliminated, leading to faster recovery of psychomotor faculties [123]. Whether (S)-ketamine is superior to racemic ketamine in the treatment of seizures warrants further study. While the benefits of ketamine in treating RSE and SRSE are promising, use of ketamine has not been widely adopted, perhaps because ketamine has not been integrated into management algorithms [178]. Therefore, integration of ketamine into treatment protocols warrants further consideration by neurologic societies and guideline creators. Furthermore, other novel uses of ketamine for seizure disorders are currently being investigated; for example, recently a case was reported in which low-dose IV ketamine was used in an epileptic patient with postoperative worsening of his seizure burden, with successful improvement in seizures and avoidance of oversedation or intubation [179]. However, it has also been recently called into question whether ketamine may induce seizure in some cases, with one recent case of new-onset seizure being reported following intramuscular ketamine administration in a pediatric patient, which certainly warrants further consideration as well [180].

There are clear benefits to early administration of ketamine for SE, including limiting the adverse events from polypharmacy and avoiding intubation [178], and earlier administration of ketamine for SE and RSE has been advocated [174, 178, 181]. Furthermore, early administration of ketamine may prevent neuronal necrosis, making it a useful medication to use early on in SE [182].

### 4.3. Ketamine and Alcohol and Substance Use Disorders

There is ongoing research surrounding the role of ketamine in treating alcohol and substance use disorders. It is thought that in addition to modulating glutamatergic neurotransmission, ketamine may mediate downstream effects on neuronal connectivity and plasticity through brain-derived neurotrophic factor and other factors to improve dopamine signaling, thereby treating drug-related synaptic deficits [183]. In a study of 111 alcohol-dependent patients, relapse rates were significantly lower at one year in patients who received intramuscular ketamine [184], though this sentinel trial lacked both randomization and blinding [185]. A study of 58 opioid-dependent patients found that subanesthetic ketamine infusion (0.5 mg/kg/hour) significantly improves immediate and short-term (48-hour) withdrawal symptoms in patients who undergo precipitated opioid withdrawal [186]. In a study of 55 cocaine-dependent patients, patients who received a single 40-minute ketamine infusion (0.5 mg/kg) in conjunction with a mindfulness-based relapse prevention program had a significantly lower relapse rate than patients who received the same mindfulness program in conjunction with a midazolam infusion [187]. Based on these promising results, it is possible that ketamine may fill a major gap in addiction treatment, as there are currently no FDA-approved medications for the treatment of cocaine use disorder [187]. Ketamine has also been shown to treat heroin dependence in a dose-dependent fashion, with one study on 70 detoxified heroin-dependent patients demonstrating that patients who received higher doses of intramuscular ketamine (2.0 mg/kg) had a significantly higher rate of abstinence at two years [188]. However, the use of ketamine in alcohol and substance use disorders is complicated by its psychotogenic, dissociative properties and conventional IV administration route, which could pose particular challenges in patients with addiction or mental illnesses [189].

## 5. Conclusions

Ketamine has emerged as a promising pharmacologic agent with diverse indications, but controversy surrounds it, as a result of its toxicities, psychedelic side effects, and abuse potential. As an antidepressant, ketamine has the benefit of being significantly faster acting than conventional agents while also having antisuicidal properties, though its long-term use is limited by toxicity and impracticality of IV infusions. In the treatment of migraines, ketamine appears to effectively reduce acute headache symptoms, while not modulating the disease state of chronic migraines. Ketamine appears to be neuroprotective and may play a role in management of TBI, subarachnoid hemorrhage, and strokes. In the treatment of pain, ketamine appears to reduce the analgesic requirement for treatment of acute pain and also has a clear role in the management of CRPS, though again its use is limited by its side-effect profile and toxicity, including neurotoxicity and memory impairment, when used long term. Ketamine may also play a role in drug detoxification and alcohol and drug relapse prevention. The role of ketamine in the management of seizures including SE and RSE is also promising. In general, ketamine has multiple nonanesthetic uses that are drawing attention, but because many studies evaluating its utility have conflicting results and sample sizes are typically small, further research studies including large-scale prospective studies are required to elucidate its role in the field of medicine.
